# Environmental impact and nutritional quality of adult diet in France based on fruit and vegetable intakes

**DOI:** 10.1007/s00394-023-03252-3

**Published:** 2023-10-06

**Authors:** Nathalie Komati, Florent Vieux, Matthieu Maillot, Nicole Darmon, Johanna Calvarin, Jean-Michel Lecerf, Marie-Josèphe Amiot, Luc Belzunces, Delphine Tailliez

**Affiliations:** 1 Agency for Research and Information on Fruit and Vegetables (APRIFEL), Paris, France; 2 MS-Nutrition, Marseille, France; 3 MoISA, University of Montpellier, CIRAD, CIHEAM-IAMM, INRAE, Institut Agro, IRD, Montpellier, France; 4 Nutrition & Physical Activity Department, Institut Pasteur de Lille, Lille, France; 5 INRAE-National Research Institute for Agriculture, Food and the Environment, Montpellier, France; 6 INRAE, Laboratory of Environmental Toxicology, UR 0406 A&E, Avignon, France

**Keywords:** Diet, Fruit and vegetables, Nutritional quality, Environmental impact, Sustainable footprint

## Abstract

**Purpose:**

To describe the nutritional quality and environmental impact of self-selected diets of adults in France in relation to their fruit and vegetable (FV) intakes.

**Methods:**

Estimates of food and nutrient intakes were taken from the national INCA3 Survey on food intakes carried out in France in 2014–2015. The population (*n* = 2121 adults) was split into five quintiles of FV intakes, in g/d (Q1 representing the lowest intake, and Q5 the highest). The nutritional quality of diets was assessed through 4 indicators: mean adequacy ratio (MAR), solid energy density, mean excess ratio (MER) and Programme National Nutrition Santé guideline score 2 (PNNS-GS2). The environmental impacts were measured with environmental footprint (EF) scores and 4 additional indicators: climate change, ozone depletion, fine particulate matter and water use. Indicators were compared between quintiles. Analysis was conducted on diets adjusted to 2000 kcal.

**Results:**

MAR and PNNS-GS2 increased with increased FV quintiles, while solid energy density decreased. Fibre, potassium, vitamin B9 and vitamin C densities increased with increasing FV intakes. Climate change, ozone depletion and fine particulate matter impacts of diets decreased with increasing quintiles of FV consumption. Conversely, water use impact increased.

**Conclusion:**

Higher intake of FV is associated with higher nutritional quality of diets and lower environmental impact, except for water use. Given the benefits of fruit and vegetables for human health and the environment, their negative impact on water use could be improved by working on the agricultural upstream, rather than by changing individuals’ food choices and reducing their consumption.

**Supplementary Information:**

The online version contains supplementary material available at 10.1007/s00394-023-03252-3.

## Introduction

The Food and Agriculture Organization of the United Nations (FAO) has defined sustainable diets as those “diets with low environmental impacts which contribute to food and nutrition security and to healthy life for present and future generations. Sustainable diets are protective and respectful of biodiversity and ecosystems, culturally acceptable, accessible, economically fair and affordable; nutritionally adequate, safe and healthy; while optimizing natural and human resources” [1]. This definition clearly shows that all these goals need to converge to ensure sustainable diets. As a result, it is clear that we need to change our diets to accommodate this transition, which could be achieved by including more healthy plant-based foods in our diets, and by increasing the intake of fruit and vegetables (FV) [2]. This transition has been recognised in numerous studies aimed at improving sustainability [3–5].

The health benefits of FV consumption for humans have been widely explored, with many studies confirming their role in the prevention of several chronic diseases, such as cardiovascular diseases [6] and certain types of cancer [7], as well as premature mortality [6]. These benefits are mainly associated with their low energy density and high nutrient densities in fibre, minerals, bioactive substances like vitamins and polyphenols, and other phytonutrients that are beneficial for the body. Moreover, insufficient intake of FV is one of the top five risk factors for health. According to the Global Burden of Disease, in 2017, about 3.9 million deaths were caused by low FV consumption [8]. Therefore, the World Health Organization (WHO) recommends a minimum daily intake of 400 g of FV to prevent the risk of chronic disease and ensure an adequate daily intake of dietary fibre [9]. However, the consumption of FV is still insufficient worldwide. In 2019, it was reported that 33% of the European population (≥ 15 years of age) did not consume any fruit or vegetables daily, and only 12% consumed the recommended minimum of 5 servings daily [10]. In France, the WHO guideline translates at the national level to a recommended consumption of at least 5 daily servings of FV (1 serving = 80–100 g) [11], but only 19.5% of the population (≥ 15 years of age) meet this recommendation [10]. According to the third French Individual and National Food Consumption Survey (INCA3) for the years 2014–2015, approximately 60% of adults in France have an average consumption below the international and national recommendation (minimum daily intake of 400 g of FV).

Beyond the benefits of FV to human health, the FAO stated in a recent report that meeting dietary guidelines (e.g., at least 400 g/d of FV) is not sufficient to reach environmental targets, and recommended reduced consumption of animal products while increasing amounts of plant-based foods to limit greenhouse gas emissions (GHGE), compared to the current diet [12]. The findings of this report are supported by a recent review showing that transitioning to plant-based diets, that focus on plant-derived foods and exclude or limit most or all animal products, could potentially reduce diet-related land use by 76%, diet-related GHGE by 49%, eutrophication by 49%, and green and blue water use by 21% and 14%, respectively [13].

All dietary patterns proposed for combined nutritional and environmental qualities (EAT-Lancet, Mediterranean diet, The New Nordic Diet, and Atlantic Diet) share the common characteristic of having a high proportion of plant-based foods, in particular significant quantities of FV [5, 13–17].

However, FV consumption, like most agricultural products, is also associated with environmental impacts. In particular, when expressed per 100 kcal instead of 100 g, FV have the highest impact of plant-based products in terms of GHGE, with impact levels similar to dairy products: 0.29 kg CO_2_ eq/100 kcal of FV versus 0.28 kg CO_2_ eq/100 kcal of dairy [18].

The aim of this study was to explore the nutritional quality and environmental impacts of self-selected diets of adults in France in relation to FV intakes. Nutritional quality was assessed using four general indicators, while environmental impact was measured using the single environmental footprint score (EF score) and four additional indicators. All indicators were compared by quintiles of daily FV consumption.

## Materials and methods

### Databases

Estimates of food and nutrient intakes were taken from the third French Individual and National Food Consumption Survey (INCA3) for 2014–2015, a cross-sectional survey carried out by the French Agency for Food, Environmental and Occupational Health & Safety (ANSES) between February 2014 and September 2015 among a representative sample of individuals living in mainland France [19]. Briefly, dietary data were self-reported through 3 non-consecutive 24-h dietary recalls consisting of 2 weekdays and 1 weekend day spread over 3 weeks. After excluding children (< 18 years of age), the final sample was composed of 2121 adults. Socio-demographic information was collected for each individual, including age class, socio-professional category (SPC), level of education, income per consumption unit (CU), physical activity level and body mass index (BMI). Three age classes were provided by INCA3 [18, 45, 65] and ≥ 65. Following the International Standard Classification of Occupations [20], SPC was divided into “low” (mainly office and manual workers), “medium” (mainly craftspeople, company directors/owners and other intermediate professions) and “high” (mainly executives and self-employed professionals) and a fourth class, labelled as “not working”, including retired, used to work, students and housewives/househusbands. Level of education was divided into 4 classes: “primary & middle school”, “high school”, “1 to 3 years of post-secondary education” and “4 or more years of post-secondary education”. Income per CU was divided into 4 classes: “ < 900 €/month/CU”, “[900–1340[ €/month/CU”, “[1340–1850[ €/month/CU” and “ >  = 1850 €/month/CU”. Physical activity level was estimated through an adapted version of the Recent Physical Activity Questionnaire [21] and considered into 3 classes: “low”, “moderate” and “high”. BMI classes (“underweight”, “normal”, “overweight” and “obese”) were derived following the WHO definition [22]. For each mixed dish reported as consumed in the dietary survey, an average recipe was estimated, based on an existing recipe database [19], and on recipes sourced from the most popular cooking website in France (i.e., marmiton.org). Based on average recipes, the amount of each specific FV was collected, and amounts of all FV within each mixed dish were summed up to estimate the proportion of FV in each mixed dish. The nutrient contents of all foods and beverages consumed were extracted from the 2016 food composition database from the French Information Centre on Food Quality [23] and matched to INCA3 dietary data for individual nutrient intake estimations. Free sugar contents of foods were estimated using a previously published procedure [24]. Foods consumed in INCA3 were categorised into 44 food categories, which were assembled into 10 major food groups for the present analysis (Supplemental Table 1). Environmental impacts of foods were extracted from Agribalyse V3.1, a life cycle assessment (LCA) database of 2520 food items published by the French Agency for Ecological Transition [25]. Details of the methodology are available elsewhere [26]. Briefly, all transportation (except from retail to consumer) and waste (except for that generated at the consumer’s place of consumption) were taken into account. The origin of the main raw materials was defined by crossing data from technical institutes and experts’ knowledge. Agribalyse was matched to dietary data at the food item level for individual environmental impact estimations. For this purpose, automated matching based on the food label was applied and verified manually. Additional information regarding cooking method, physical characteristics (e.g., “reconstituted from powder” for coffee), etc., were taken into account to further improve the matching. Consumed foods that were not sufficiently described in Agribalyse were matched to average values (e.g., a consumed “potato” was matched to a weighted average item of different potatoes). Remaining unmatched foods were manually matched to their best alternative (e.g., consumed food “daikon” was matched to the environmental impacts of “black radish”).

## Indicators of nutritional quality

The nutritional quality of individual diets was assessed through 4 indicators: mean adequacy ratio (MAR), solid energy density, mean excess ratio (MER) and Programme National Nutrition Santé—guideline score 2 (PNNS-GS2). The MAR was calculated as the mean of 23 nutrient adequacy ratios, each corresponding to the percentage of the daily recommended intake for one of the 23 nutrients [27–29] (see Supplemental Table 2). Each nutrient adequacy ratio was truncated at 100 so that a high intake of one nutrient could not compensate for a low intake of another. MAR ranged from 0 (no nutrient intake) to 100 (coverage of all 23 recommended intakes). Solid energy density was calculated for each diet by dividing energy intake by ingested weight for solid food only. Diets with a low energy density are associated with high diet quality [30]. The MER was calculated as the mean percentage of excess sodium, saturated fatty acids (SFA) and free sugars [18]. Upper limits were taken from ANSES [28, 29] and WHO guidelines [33] for free sugars. The PNNS-GS2 was used to estimate adherence of diets to the French Nutrition and Health Programme recommendations, by allocating points when the diet is in accordance with the recommendation [34]. Higher PNNS-GS2 is associated with higher adherence to 2017 dietary guidelines.

## Environmental indicators

The single environmental footprint (EF) score [35], also called the product environmental footprint (PEF) score, (in mPt) was considered a general measure of the environmental impact of diet and was calculated for each individual. It is a weighted mean of 16 environmental impacts and was calculated following the environmental footprint (EF) 3.0 life cycle impact assessment method [36], as well as the adaptations proposed for Agribalyse 3.1 for four impact categories (i.e., climate change, ecotoxicity, human toxicity cancer and human toxicity non-cancer) [37]. Three additional environmental indicators that are considered the most robust and with the best level of consensus were also included in our study: a) climate change (in kg CO_2_ eq); b) ozone depletion (in E-06 kg CFC 11 eq), which increases exposure to harmful ultraviolet radiation; and c) fine particulate matter (E-06 disease incidence) that has an effect on human health via air exposure. A fourth indicator with a lower level of consensus was retained: blue water (fresh surface and groundwater used for irrigation), called here water use (in m^3^). Data from Agribalyse 3.1 are the result of over 10 years of research and expertise, aiming to provide the most accurate possible representation of the environmental impact of agricultural and food products; however, this work is constantly evolving and being improved. The limitations of the environmental impact indicators are those of the Agribalyse methodology and include a lack of representation of all biodiversity-friendly pressures and practices, a lack of description of transformation processes and the use of co-products in the food industry, as well as a spatialisation of certain impact categories, coupling impact indicators with eco-system service indicators. This means that when life cycle analysis is used to compare the environmental impacts of different production systems (organic versus conventional farming, intensive/extensive livestock rearing, etc.) and processing, these limitations must be explicitly highlighted.

### Statistical analysis

FV consumption was estimated for each individual (*n* = 2121). To estimate the daily quantity of FV, all fruit (including dried fruits, but not nuts) and vegetables consumed, including those in mixed dishes, were counted. In accordance with French national guidelines, fruit juices (100% pure juice) were counted only up to one glass (125 g). Individuals were split into quintiles according to their daily FV consumption, in g/day (Q1 the lowest, Q5 the highest). Associations between socio-demographic characteristics and FV consumption quintiles were tested using Chi^2^ tests, and energy intakes were compared between quintiles of FV consumption using analysis of variance (ANOVA). As energy intakes could be different from one quintile to another, each diet was proportionally adjusted to a theoretical average adult daily energy intake of 2000 kcal to control for inter-individual differences in energy intakes and compare quintiles of FV consumption independently of energy intake levels. The contributions of the 10 major food groups to 2000 kcal diets were estimated and compared between quintiles of FV consumption.

Means of environmental impacts (EF, climate change, ozone depletion, fine particulate matter and water use) and nutritional quality (MAR, solid energy density, MER, and PNNS-GS2) indicators, as well as nutrient intakes (expressed in percentage of dietary reference value—DRV), were statistically compared between quintiles of FV consumption. Then, trend tests across quintiles and 2-by-2 comparison tests between quintiles using Bonferroni correction (Supplemental Table 3) were conducted. For environmental indicators, total impacts were expressed as a percentage of the most impacting quintile. Contributions within each quintile were then expressed as a percentage of the total contribution. Statistical analyses were computed using the SURVEYREG (to compare quantitative indicators between quintiles) and SURVEYFREQ (for qualitative socio-demographic variables) procedures of SAS Statistical Software (version 9.4) to ensure national representativeness. I threshold for statistical significance was *p* < 0.05.

## Results

Mean FV consumption ranged from 119 g/d in Q1 (111.3–125.8) to 694 g/d in Q5 (672.6–714.6) (unadjusted amounts, Table 1). Age increased with increasing quintiles of FV intake (74.9% of 45 yo or more in Q5 against 41.4% in Q1), but no clear trend was observed for the gender ratio. Physical activity levels significantly increased across FV quintiles, but no relation was observed with BMI status. All three socio-economic variables, namely SPC, income per consumption unit and educational level, were significantly and positively associated with the level of FV consumption. The relationship with income was very strong, with 40% of individuals in the lowest FV quintile belonging to the lowest socio-economic class (i.e., living in a household with < 900 €/month/CU), and only 15% of individuals in the highest quintile of FV intakes belonging to this class.

**Table 1:** Daily FV consumption, daily energy intake and socio-demographics characteristics of individuals among the five quintiles of FV consumption

		Q1	Q2	Q3	Q4	Q5	*p*-value
Amounts of FV (g/d) 95% CI		118.6 111.3–125.8	243.4 238.4–248.3	355.9 352.0–359.8	465.7 461.1–470.2	693.6 672.6–714.6	< 0.001
Energy intakes (kcal/d) 95% CI		1894 1739.1–2048.25	2065 1934.0–2195.5	2110 2012.1–2207.6	2167 2065.5–2267.6	2277 2183.0–2370.2	0.0014
Gender	Men	44.8	46.7	45.7	50.9	54.3	0.3289
	Women	55.2	53.3	54.3	49.1	45.7	
Age	18.0–44.0 year-old	58.6	57.1	50.2	38.6	25.2	< 0.001
	45.0–64.0 year-old	32.7	28.8	33.6	40.1	47.4	
	65.0–79.0 year-old	8.7	14.1	16.2	21.3	27.5	
SPC	Low	36.1	36.1	27.9	17.2	20.9	< 0.001
	Medium	26.1	21.1	23.4	22.0	18.1	
	High	9.6	13.5	16.9	17.7	17.6	
	Not working	28.2	29.2	31.7	43.2	43.4	
Income per consumption unit	< 900 €/month/CU	40.2	25.2	19.8	23.7	15.3	< 0.001
	[900–1340[ €/month/CU	23.9	20.8	22.6	23.6	23.8	
	[1340–1850[ €/month/CU	21.5	23.7	25.2	19.6	27.0	
	> = 1850 €/month/CU	14.4	30.3	32.4	33.1	34.0	
Educational level	Primary + middle school	53.0	48.0	45.8	46.6	46.6	0.0075
	High school	23.2	21.3	19.8	12.6	15.1	
	1 to 3 years of post-secondary education	13.2	16.9	14.0	22.0	18.7	
	4 or more years of post-secondary education	10.6	13.8	20.5	18.8	19.7	
Physical activity level	Low	42.4	45.7	36.1	30.5	31.3	0.0258
	Moderate	44.6	45.5	51.9	51.7	55.2	
	High	13.0	8.8	12.0	17.8	13.6	
BMI	Underweight	3.3	5.0	2.7	2.4	1.4	0.6192
	Normal	42.9	45.4	44.9	49.3	41.8	
	Overweight	32.9	32.8	35.1	34.9	39.5	
	Obesity	20.9	16.8	17.3	13.3	17.3	

*p*-value from ANOVA for quantitative variables and Chi^2^ test for qualitative ones

*SPC* socio-professional category, *CU* consumption unit, *BMI* body mass index

As expected, energy intakes increased significantly from Q1 (1894 kcal/d) to Q5 (2277 kcal/d). As indicators of nutritional quality and environmental impact are correlated with energy intakes [18], the subsequent analyses were conducted on diets adjusted to 2000 kcal. However, absolute (i.e., unadjusted) daily quantities and environmental impacts in each quintile are available in Supplemental Table 4.

**Fig. 1:**
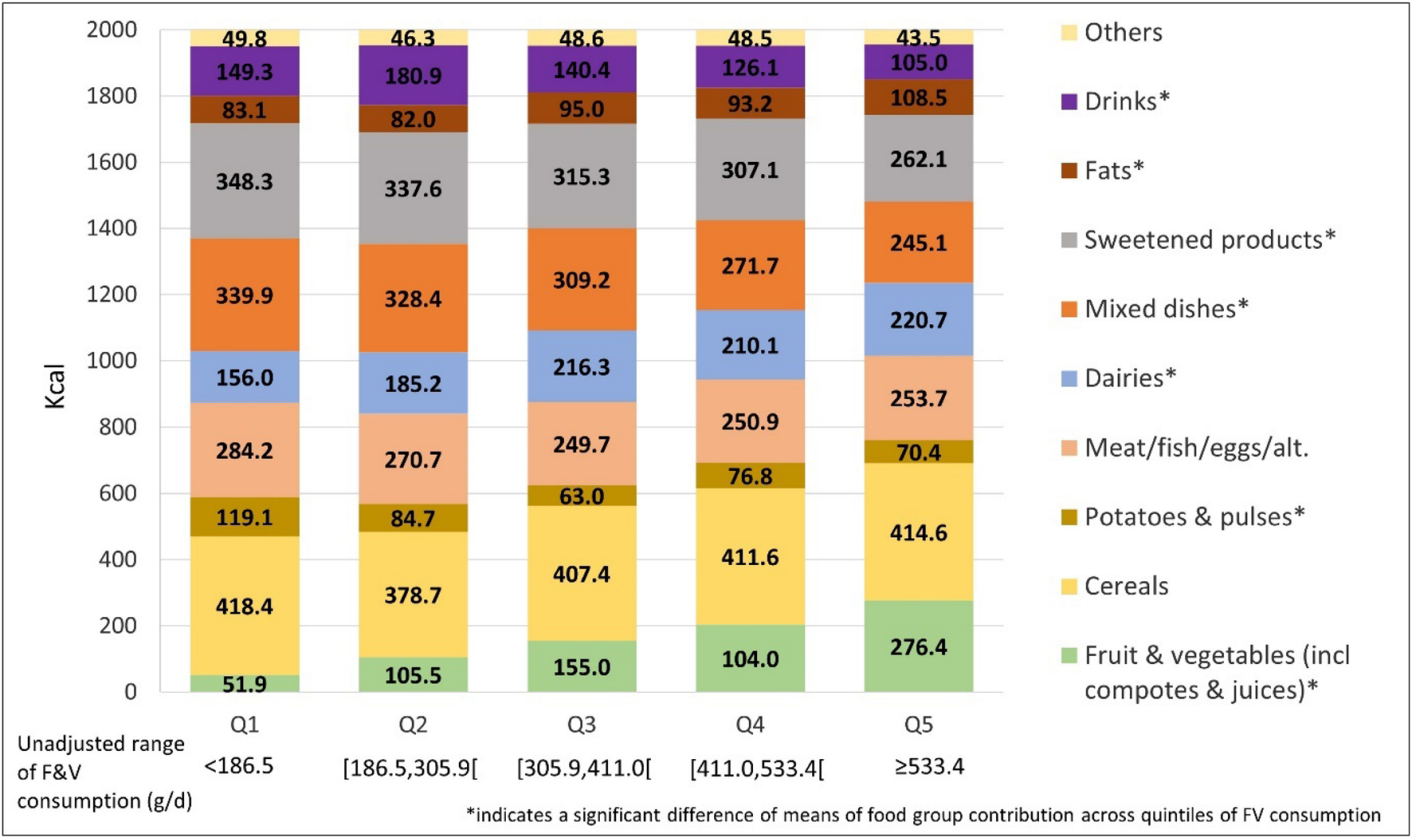
Mean energy provided by the different food groups to diet adjusted to 2000 kcal according to quintiles of FV consumption

Figure 1 shows the mean energy provided by the different major food groups to diets adjusted to 2000 kcal in each quintile of FV intake. Increased quantities of FV from Q1 to Q5 were associated with increased energy contribution from FV. This increased energy contribution of FV was associated with increased energy contribution from "dairies” and “fats”, and with decreased energy contributions from “sweetened products”, “drinks”, and “mixed dishes” as well as from “potatoes and pulses”. In contrast, the mean energy contributions from “cereals” and from the “Meat, fish, eggs and alternatives” group were not significantly different across quintiles of FV consumption.

**Fig. 2:**
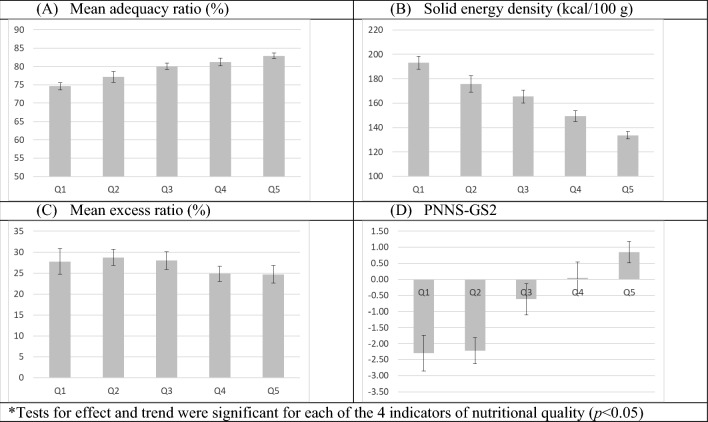
Mean (95% CI) nutritional quality of diets adjusted to 2000 kcal according to quintiles of FV consumption*

Figure 2 shows the indicators of nutritional quality for diets adjusted to 2000 kcal according to quintiles of FV intake. As FV consumption increased, MAR and PNNS-GS2 increased, while solid energy density decreased (*p* < 0.05). MER also decreased (*p* < 0.05), but analysis of the differences quintile by quintile (Supplemental Table 3) showed that none of them were significant.

Table 2 shows the four nutritional quality indicators, nutrient contents (expressed in % of DRV) and environmental impacts of diets adjusted to 2000 kcal in the five quintiles of FV intakes. Increased consumption of FV was characterised by increasing densities in fibre, EPA + DHA, potassium, vitamin A, vitamin E, vitamin B9 and vitamin C (significant 2-by-2 comparisons between all quintiles). Among nutrients to limit (SFA, sodium, free sugars), free sugar intakes were the only one that progressively decreased with increased FV consumption. However, 2-by-2 significances were observed only with Q5 as a reference against Q1, Q2 and Q3, but not Q4. There were no differences in proteins, linoleic acid, alpha-linolenic acid, vitamins B12 and D, iron, magnesium and zinc densities between quintiles. EF impacts of diets adjusted to 2000 kcal reached 0.710, 0.711, 0.690, 0.702 and 0.681 mPt from Q1 to Q5, respectively. The difference was not significant (*p* = 0.5302). The effect of quintiles on other environmental impact indicators was significant.

**Table 2:** Mean nutritional characteristics and environmental impacts of diets adjusted to 2,000 kcal according to the quintiles of FV consumption

	Q1	Q2	Q3	Q4	Q5	*p*-value	*p*-trend
Nutritional quality indicators							
MAR, % adequacy	74.6	77.2	80.1	81.24	82.9	< 0.001	< 0.001
MER, % excess	27.8	28.7	28.0	24.85	24.7	0.010	0.016
Solid energy density, kcal/100 g	193.1	175.8	165.4	149.37	133.7	< 0.001	< 0.001
PNNS-GS2	− 2.3	− 2.2	− 0.6	0.1	0.8	< 0.001	< 0.001
Nutrients (in % of DRV*)							
Nutrients to favour							
Proteins	172.2	172.4	168.7	168.3	165.7	0.563	0.126
Fibres	51.9	57.2	62.4	70.0	78.4	< 0.001	< 0.001
Linoleic acid	81.3	78.5	81.0	76.6	78.3	0.542	0.259
Alpha linolenic acid	41.4	43.2	45.6	45.4	47.9	0.094	0.008
EPA + DHA	44.1	59.2	60.6	60.8	68.5	0.001	0.001
Vitamin A	85.3	126.6	133.0	141.5	169.6	< 0.001	< 0.001
Vitamin B1	139.3	151.0	152.8	151.0	157.8	0.004	0.013
Vitamin B2	103.0	105.4	112.9	109.1	112.5	0.033	0.008
Vitamin B3	169.0	160.8	151.0	154.7	151.8	0.027	0.002
Vitamin B6	96.1	96.9	101.1	106.0	110.4	< 0.001	< 0.001
Vitamin B9	72.1	81.4	92.7	99.4	109.4	< 0.001	< 0.001
Vitamin B12	133.5	131.1	136.8	129.3	136.0	0.970	0.922
Vitamin C	42.5	62.3	84.4	96.8	126.6	< 0.001	< 0.001
Vitamin E	90.4	92.5	109.2	103.5	116.0	< 0.001	< 0.001
Vitamin D	18.8	20.9	21.6	21.8	21.8	0.082	0.010
Calcium	87.3	92.7	100.9	97.5	98.6	0.001	< 0.001
Potassium	76.6	80.2	84.3	89.8	97.4	< 0.001	< 0.001
Iron	72.1	72.6	73.1	74.3	76.4	0.383	0.096
Magnesium	98.3	97.7	99.4	101.5	104.2	0.172	0.024
Phosphorus	211.1	212.2	219.4	220.4	220.3	0.033	0.003
Zinc	92.4	93.5	89.0	88. 6	85.8	0.187	0.052
Copper	90.3	93.7	94.2	100.6	108.4	0.001	< 0.001
Iodine	90.2	90.7	100.1	98.9	104.2	< 0.001	< 0.001
Selenium	176.1	169.6	175.9	182.9	190.8	0.012	0.019
Nutrients to limit							
Saturated fatty acids	121.4	122.4	121.4	118.5	114.1	0.012	0.012
Sodium	126.2	133.2	134.9	133.6	140.5	0.004	< 0.001
Free sugars	109.8	105.5	100.9	95.8	85.2	< 0.001	< 0.001
Environmental impact indicators							
EF Score, mPt	0.7	0.7	0.7	0.7	0.7	0.530	0.199
Climate change, kg CO2 eq	5.6	5.6	5.3	5.3	5.0	0.011	0.003
Ozone depletion, E-06 kg CFC 11 eq	0.7	0.7	0.7	0.6	0.6	0.008	< 0.001
Fine particulate matter, E-06 disease incidence	0.6	0.6	0.5	0.5	0.5	0.014	0.001
Water use, m^3^	6.0	6.6	7.8	8.9	9.1	< 0.001	< 0.001

^*^Dietary references values [27–29, 38]

**Fig. 3:**
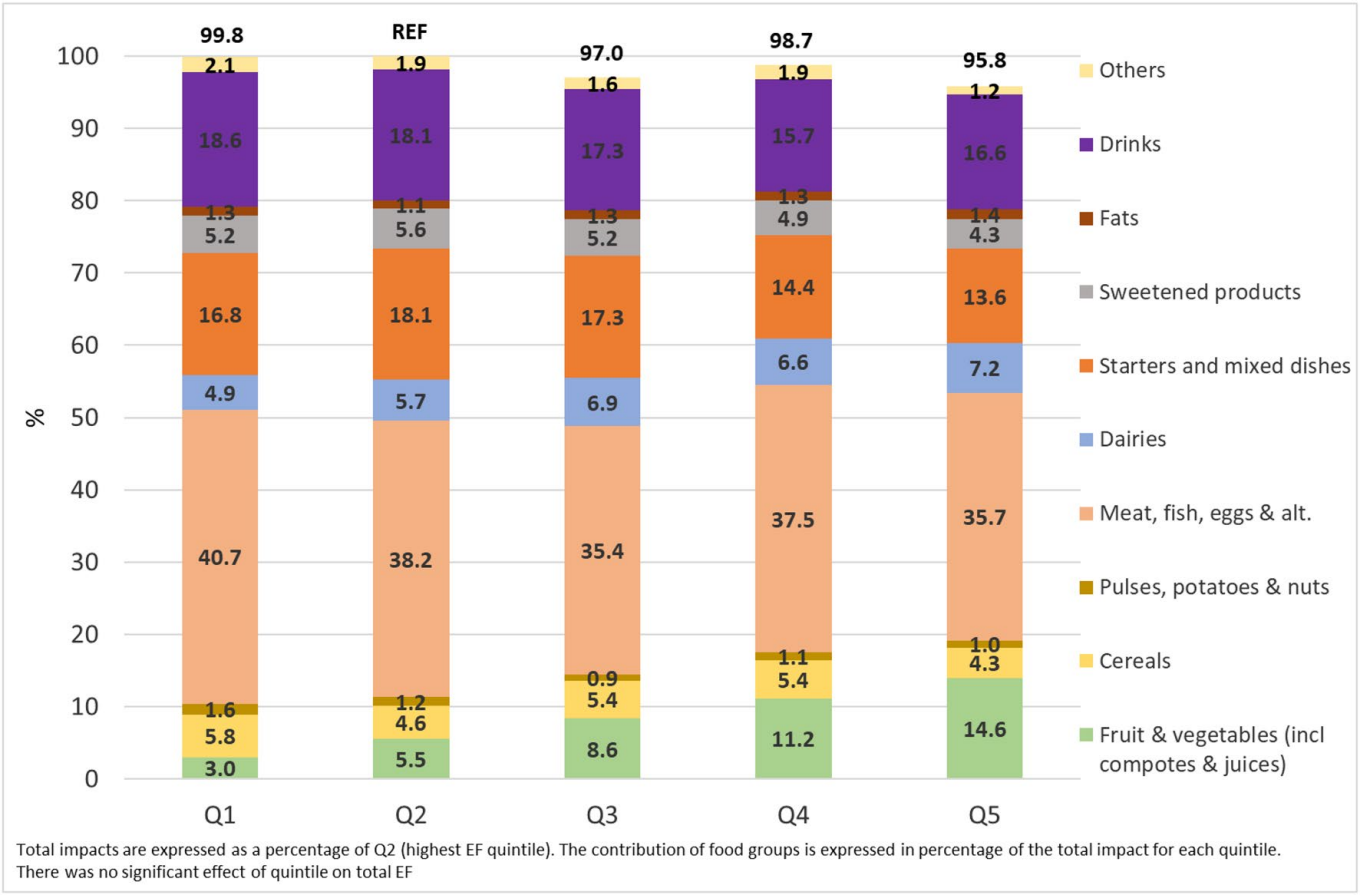
Mean contribution of food groups to single EF score of 2000 kcal adjusted diets according to quintiles of FV consumption

In Fig. 3, Q2 having the highest value of EF impact, has been selected as a reference (i.e., 100), and total EF impacts from other quintiles were expressed as a percentage of this reference. Within each quintile, contributions of food groups were expressed as a percentage of the quintile’s total. The food group “Meat, fish, eggs and alternatives” was the major contributor to the EF impact of diets (between 35 and 41%) in all quintiles.

**Fig. 4:**
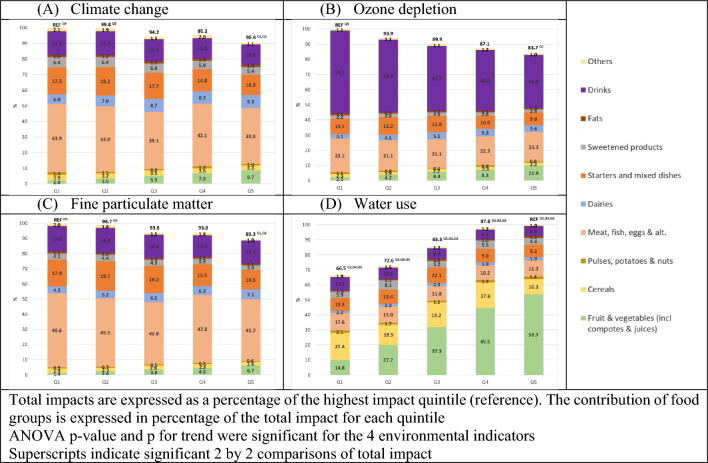
Mean contribution of food groups to impacts on climate change, ozone depletion, fine particulate matter and water use of diets adjusted to 2000 kcal according to quintiles of FV consumption

Figure 4 shows that impacts on climate change, ozone depletion and fine particulate matter from diets adjusted to 2000 kcal decreased with increasing quintiles of FV consumption. Conversely, impacts on water use in diets adjusted to 2000 kcal increased with increasing quintiles of FV consumption, with, in Q5 a contribution to this impact from FV of 54%. Fresh fruit alone accounted for 2.72 m^3^/2000 kcal in Q5, corresponding to 30% of water use associated with the entire diet adjusted to 2000 kcal (data not shown). The food group “Meat, fish, eggs and alternatives” was the major contributor to climate change (between 39 and 44%) and fine particulate matter (between 44 and 49%), while “drinks” was the major contributor to ozone depletion (between 42 and 55%), mainly due to bottled water and cold non-alcoholic beverages (data not shown).

## Discussion

To our knowledge, this is the first study to simultaneously assess the nutritional quality and environmental impacts of self-selected diets in relation to levels of FV consumption. Diets were adjusted to 2000 kcal (theoretical average adult energy intake), which allows for standardisation and to have a homogeneous functional unit [39].

Our results revealed that the lowest consumers of FV (Q1) have an average intake of 119 g/d, which corresponds to less than 2 daily servings of FV. These low consumers are more likely to be young and to have a lower socio-economic level than the highest-level consumers. The most recent National Diet and Nutrition Survey in the United Kingdom (NDNS, 2020) showed comparable results, with 67% of adults surveyed reporting consumption of less than the quantities recommended in guidelines [40]. These observations are consistent with the situation observed in 2019 in Europe, with 88% of the population aged 15 years and over not meeting the recommendation [41].

We observed that nutritional quality increased systematically with increasing intakes of FV. Hence, high FV intakes were characterised by lower energy density and higher densities of vitamins C (126.6% in Q5 vs 42.5% in Q1), B9 (109.4% in Q5 vs 72.0% in Q1), potassium (97.4% in Q5 vs 76.6% in Q1) and fibre (78.5% of DRVs in Q5 vs 52.0% of DRVs in Q1), contributing to a higher MAR, and lower amounts of free sugars compared to groups consuming less FV. This finding is supported by data from the literature showing that FV contains high amounts of a wide range of beneficial nutrients including fibre, vitamins (A, B9 and C), and minerals such as potassium [42, 43]. In addition, higher consumption of FV was associated, in our study, with higher densities of nutrients that are not found in FV, such as nutrients that are specific to fish products (iodine and EPA + DHA) and to dairies (calcium), without reducing densities of nutrients specific to meat products (iron, vitamin B12), showing that an increased consumption of FV is associated with a more balanced diet.

Our results also showed that as FV consumption increased, MER decreased, showing the importance of increasing FV intake. However, this raises the question of substitution when increasing intake. According to our findings, the contribution of cereals and meat/fish/eggs remained similar in the different quintiles, which could allow us to suggest that the FV pattern is independent of the meat/egg/fish and cereal patterns.

Although our results showed an association between higher FV consumption and higher fibre intake, ranging from 51.95% of DRVs for Q1 to 78.45% of DRVs for Q5, the recommended intakes were not met in adults as mean intakes remained below DRVs in all quintiles. A review conducted in 2017 reported comparable results for adults in Europe, showing that fibre intake was below the recommendations. Grain products, especially bread, have been identified as the primary food source of fibre, while vegetables, potatoes and fruits are secondary fibre contributors, providing 12.0% to 21.0%, 6.0% to 19.0% and 8.0% to 23.0% of fibres, respectively [43].

Regarding the environmental impact of the studied diet, there were no significant differences in single EF scores between quintiles of FV consumption, showing that the level of FV consumption is independent of environmental impact when estimated with an aggregate indicator. This finding could be explained by the low contribution of FV to the single EF score, compared to other food groups. FV intake (excluding that from mixed dishes) contributed between 3% (Q1) to 14.6% (Q5) of the total single EF score, whereas the major contributors were meat products with 35.4% (Q3) to 40.7% (Q1) of the total. This finding is confirmed by EAT-Lancet [14] and World Wildlife Fund [44] studies, according to which meat and dairy food groups are the highest contributors to single EF scores, but also to other environmental impacts such as climate change, ozone depletion or fine particulate matter.

When the environmental indicators were analysed separately, higher FV consumption was associated with higher impact on water use compared to other food groups, but had less impact on climate change, fine particulate matter and ozone depletion, even for the highest consumers of FV (Q5). The low contribution of FV to the total impact (single EF score) compared to other food groups, and the characteristics of the French market (half of FV are produced in France, with a low amount of inputs and fertilisation in production) [45, 46] could explain their low impact on climate change, fine particulate matter and ozone depletion. Our results suggest that increasing the intake of FV at constant energy intake will have little impact on climate change, fine particulate matter and ozone depletion, but may increase water use impact. The contribution of FV should, therefore, be considered for reducing water use, although meat consumption remains the priority to reduce the environmental impacts of diet [16, 47].

A recent study assessed the environmental impact of diets in Sweden for six indicators (GHGE, cropland use, nitrogen (N) and phosphorus (P) application, consumptive water use and extinction rate). In this study, animal-based foods contributed most to the total environmental impact of diet (23.0–83.0%), followed by plant-based foods (8.0–40.0%), and discretionary foods (9.0–37.0%) for all environmental indicators. While animal-based foods contributed mainly to GHGE, cropland use, and N and P application, plant-based and discretionary foods had more significant impacts on consumptive water use and extinction rates (together responsible for 70–77% of the total dietary impact on these indicators) [48]. Comparable results were also observed in a recent population-based study conducted in Israel, where meat was the major contributor to land use, dairy to GHGE, and fruits followed by vegetables to water use; however, the authors highlighted that most FV in this country are grown using treated wastewater, which could reduce pressure on the environment [49].

### Limitations, strengths, and challenges

The main strength of the present study was the matching of Agribalyse, the official national database on environmental impacts, with the INCA3 database, representative of all individuals residing in metropolitan France (excluding Corsica) and living in an ordinary household. In addition, INCA3 respects the methodology recommended by the European Food Safety Authority (EFSA) [50]. However, the INCA3 study, like all studies assessing dietary intakes, is based primarily on self-reporting by participants, which makes the reliability of the data partly dependent on the cognitive abilities of the participants, and on possible biases in reporting. Another limitation is the uncertainty in extrapolating long-term consumption from short-term cross-sectional, dietary reports [51]. Also, food consumption data from INCA3 were last updated in 2014–2015, whereas the food consumption habits of the French population may have changed since then, specifically due to the COVID-19 pandemic and inflation [52–58].

Our study also has several limitations and challenges that need to be addressed. It was impossible to dissociate the impacts of the ingredients of mixed dishes. Recipes indicating the amounts of ingredients for each meal enabled us to quantify the total amounts of FV for each individual. However, nutritional composition or environmental impacts were only available for the final dish and not for each ingredient, which made it impossible to assess the nutritional intake or environmental impacts of total FV consumption (i.e., including FV from mixed dishes).

Assessing the environmental performance of diets requires a multicriteria environmental perspective, going beyond climate change impacts alone [59]. In addition, optimal diets remain complex to model due to the many types of food products consumed, and the diversity of agricultural production systems, supply chains and local environmental settings. The majority of LCA databases are not exhaustive and lack data regarding the diversity of productions [59, 60]. Environmental data on FV production and imports from Agribalyse are generic estimates as not all crops are assessed [61, 62]**,** which could impair the robustness of our results. However, LCA data were used for comparative purposes here and for the 2-by-2 comparisons, thus reducing the risk of over-interpretating absolute values that may suffer of uncertainties.

In addition, our results showed that not all environmental indicators behave similarly, confirming that it is essential to adopt a multicriteria assessment approach to better understand the environmental impact of diet.

Regarding the water use indicator, it would be necessary to approach this indicator more precisely, particularly by better taking into account spatial and temporal variability in LCA inventories and impact calculations [63]. Nevertheless, the results obtained on the overall dietary contribution to environmental impacts illustrate the importance of focusing on water use when adopting a more plant-based diet. This indicator has rarely been addressed, with scientific studies mainly focusing on GHGE and land use [16]. Linking the real contribution of adopting a more plant-based diet to water use is a short-term challenge, as global warming will make access to water resources more difficult and may potentially decrease FV consumption and thus disrupt the human health benefit/planetary health risk balance of FV. Production modes with irrigation techniques and territorial management should also be considered in the evaluation of water use, in order to identify pathways to reduce use of this resource. In fact, the efficiency of crop water use could be increased through irrigation strategies based on physical models of evaporation from partially wetted soil surfaces, irrigation water redistribution in the soil, and root water uptake. Micro-sprinkling seems to be the most suitable irrigation technique for efficient use of water, as it combines the advantages of drip irrigation with the capacities of sprinkler irrigation [64]. Other farm management practices will also play a key role in efficient water use, such as understanding soil types and structures, the need for water depending on the season and the development cycle of crops, keeping the soil covered with living or organic mulch to retain moisture, and selecting drought-tolerant crop species and varieties [65].

## Conclusion

For the vast majority of adults in France, FV consumption remains insufficient to achieve optimal health. This study showed that, compared to individuals with low intakes of fruit and vegetables, those with higher intakes have diets of better nutritional quality and lower environmental impact for all the indicators included, except for water use. Given the benefits of FV for human health and the environment, increasing their proportion in diets is essential and should be a national priority in the framework of balanced diets and nutrition. Regarding their negative impact on water use as observed in our study, this could be mitigated by working on the agricultural upstream rather than by reducing their consumption, such as working on the choice of crop varieties and production methods that better preserve resources to benefit from the nutritional advantages of FV without increasing the negative impacts on water use. The desirability of consuming products with the most water-efficient production systems was illustrated in our study (e.g., micro-irrigation for tomatoes, etc., or from agricultural areas under little pressure on water resources). An information and promotion campaign for these production methods could be considered to facilitate consumer choice. Increasing consumption of these products could thus make a major contribution to a sustainable food system. Additional research is needed to better take into account production practices that enable reduced field emissions and water use impacts.

### Supplementary Information

Below is the link to the electronic supplementary material.

Supplementary file1 (DOCX 70 kb)

## Data Availability

Data will be made available upon reasonable request.
