# Supplementary material for: Environmental impact and nutritional quality of adult diet in France based on fruit and vegetable intakes
Source: Eur J Nutr. 2023 Oct 6;63(1):195–207. doi: 10.1007/s00394-023-03252-3 (PMC10799092; doi:10.1007/s00394-023-03252-3)
Supplement: Supplementary file 1 — Supplementary file1 (DOCX 70 kb) [file 394_2023_3252_MOESM1_ESM.docx]

Supplemental data:

Supplemental Table 1. Food categorisation

| Refined bread and dried bread products | Cereals | "Rusk White/refined", "Crisp bread, wheat, refined flour" |
| --- | --- | --- |
| Whole-grain or semi-whole-grain bread and dried bread products |  | "Rusk, wholemeal", "Rye bread, wholemeal" |
| Breakfast cereals and cereal bars |  | "Cereal bars plain", "Processed wheat-based flakes" |
| Refined pasta, rice, wheat and other cereals |  | "Pasta", "Rice grain" |
| Whole-grain and semi-whole-grain pasta, rice, wheat and other cereals |  | "Pasta wholemeal","Rice grain, Integral /not refined" |
| Vegetables | Fruits, Vegetables and legumes | "Sweet peppers", "zucchini" |
| Pulses |  | "Lentils", "Canned or jarred chickpea" |
| Potatoes and other tubers |  | "Potato boiled", "Pan-fried potato" |
| Fresh and dried fruit |  | "Apples", "Dried apricots" |
| Fruit purées and fruits in syrup |  | "Fruit compote", "Canned or jarred pineapple" |
| Seeds and nuts |  | "Walnuts", "Almonds" |
| Milk | Dairies | "Cow milk", "Cow milk, Low / reduced lactose" |
| Yoghurt and fromage blanc |  | "Quark", "Yoghurt" |
| Cheese |  | "Cheese, camembert", "Cheese, edam" |
| Eggs and egg dishes | Meat, fish, eggs & alternatives | "Omelette", "Whole eggs" |
| Meat (excl. poultry) |  | "Bovine, minced meat", "Pig muscle" |
| Poultry |  | "Chicken fresh meat", "Duck fresh meat" |
| Delicatessen meats |  | "Pate", "Ham" |
| Fish |  | "Salmons", "Tuna" |
| Crustaceans and molluscs |  | "Shrimps", "Oysters" |
| Offal |  | "Veal liver", "Bovine tongue" |
| Substitutes for animal products made from soya and other plants |  | "Almond drink", "Tofu" |
| Croissant-like pastries, pastries, cakes and sweet biscuits | Sweetened products | "Brioche type products", "Sponge cake" |
| Dairy-based and cream desserts |  | "Custard", "Milk rice" |
| Ice cream, frozen desserts and sorbet |  | "Ice cream", "Sorbet" |
| Confectionery and chocolate |  | "Milk chocolate", "Hard candies" |
| Sugar and sweeteners |  | "Jam", "White sugar" |
| Animal fats | Fats | "Butter", "Crème fraiche" |
| Vegetable fats |  | "Olive oils", "Margarines" |
| Soups and broths | Starters and mixed dishes | "Mixed vegetables soup", "Gazpacho and similar" |
| Meat dishes |  | "Calf fresh meat, Carrots, Common mushrooms, White sauces", "Goulash" |
| Fish dishes |  | "Fish balls", "Fish fingers" |
| Vegetable dishes |  | "Moussaka", "Vegetables, gratinated" |
| Potato, cereal or pulse dishes |  | "Risotto", "Lasagna", |
| Sandwiches, pizzas, pies, savoury pastries and biscuits |  | "Hamburger with bread", "Pizza" |
| Bottled water | Drinks | "Carbonated bottled drinking water", "Natural mineral water" |
| Tap water |  | "Tap water" |
| Cold non-alcoholic beverages (CNABs) |  | "Cola-type drinks", "Beer, alcohol-free" |
| Fruit and vegetable juice |  | "Mixed fruit and vegetable juices", "Orange juice" |
| Alcoholic beverages |  | "Beer", "Wine" |
| Hot beverages |  | "Tea infusion (black, white)", "Coffee" |
| Condiments, herbs, spices and sauces | Others | "Basil", "Hummus" |
| Prepared dishes and desserts for infants |  | "Ready-to-eat dairy-based meal for children", "Biscuits, rusks and cookies for children" |
| Infant milks and drinks |  | "Fruit and vegetable juices and nectars specific for infants and young children", "Follow-on formula" |

Supplemental Table 2. Dietary reference values

| Nutrient | Unit | Men | Women | DRV |
| --- | --- | --- | --- | --- |
| Proteins | %E | ≥10 | ≥10 | RI |
| Fibres | g/j | 30 | 30 | AI |
| Linoleic acid | %E | 4 | 4 | PRI |
| Alpha linolenic acid | %E | 1 | 1 | PRI |
| EPA+DHA | mg/d | 500 | 500 | PRI |
| Vit A | µg ret eq/d | 750 | 650 | PRI |
| Vit B1 | mg/MJ | 0.1 | 0.1 | PRI |
| Vit B2 | mg/d | 1.6 | 1.6 | PRI |
| Vit B3 | mg/MJ | 1.6 | 1.6 | PRI |
| Vit B6 | mg/d | 1.7 | 1.6 | PRI |
| Vit B9 | µg DFE/d | 330 | 330 | PRI |
| Vit B12 | µg/d | 4 | 4 | AI |
| Vit C | mg/d | 110 | 110 | PRI |
| Vit E | mg/d | 10 | 9 | AI |
| Vit D | µg/d | 15 | 15 | AI |
| Calcium | mg/d | 950 | 950 | PRI |
| Potassium | mg/d | 3500 | 3500 | AI |
| Iron | mg/d | 11 | 16 | PRI |
| Magnesium | mg/d | 380 | 300 | AI |
| Phosphorus | mg/d | 550 | 550 | AI |
| Zinc | mg/d | 11.7 | 9.3 | PRI |
| Copper | mg/d | 1.9 | 1.5 | AI |
| Iodine | µg/d | 150 | 150 | AI |
| Selenium | µg/d | 70 | 70 | AI |
| SFA | %E | 12 | 12 | UL |
| Sodium | mg/d | 2300 | 2300 | UL |
| Free sugars | %E | 10 | 10 | UL |
| AI=adequate intake, PRI=population reference intake, UL=upper limit, RI=reference interval | | | | |

Supplemental Table 3. *p*-values of 2-by-2 comparison tests using Bonferroni correction

|  | 1vs2 | 1vs3 | 1vs4 | 1vs5 | 2vs3 | 2vs4 | 2vs5 | 3vs4 | 3vs5 | 4vs5 |
| --- | --- | --- | --- | --- | --- | --- | --- | --- | --- | --- |
| MAR | 0.055 | <0.001 | <0.001 | <0.001 | 0.007 | <0.001 | <0.001 | 1.000 | <0.001 | 0.149 |
| Solid energy density | <0.001 | <0.001 | <0.001 | <0.001 | 0.198 | <0.001 | <0.001 | <0.001 | <0.001 | <0.001 |
| MER | 1.000 | 1.000 | 0.974 | 0.997 | 1.000 | 0.062 | 0.099 | 0.244 | 0.214 | 1.000 |
| PNNS-GS2 | 1.000 | <0.001 | <0.001 | <0.001 | <0.001 | <0.001 | <0.001 | 0.595 | <0.001 | 0.069 |
| Proteins | 1.000 | 1.000 | 1.000 | 1.000 | 1.000 | 1.000 | 0.997 | 1.000 | 1.000 | 1.000 |
| Fibres | 0.003 | <0.001 | <0.001 | <0.001 | 0.005 | <0.001 | <0.001 | <0.001 | <0.001 | <0.001 |
| Linoleic acid | 1.000 | 1.000 | 1.000 | 1.000 | 1.000 | 1.000 | 1.000 | 1.000 | 1.000 | 1.000 |
| Alpha linolenic acid | 1.000 | 1.000 | 1.000 | 0.103 | 1.000 | 1.000 | 0.217 | 1.000 | 1.000 | 1.000 |
| EPA+DHA | 0.300 | 0.422 | 0.060 | 0.003 | 1.000 | 1.000 | 1.000 | 1.000 | 1.000 | 1.000 |
| Vit A | 0.002 | <0.001 | <0.001 | <0.001 | 1.000 | 1.000 | 0.005 | 1.000 | 0.002 | 0.317 |
| Vit B1 | 0.382 | 0.093 | 0.170 | 0.005 | 1.000 | 1.000 | 1.000 | 1.000 | 1.000 | 0.404 |
| Vit B2 | 1.000 | 0.188 | 1.000 | 0.131 | 0.325 | 1.000 | 0.272 | 1.000 | 1.000 | 1.000 |
| Vit B3 | 1.000 | 0.082 | 0.157 | 0.019 | 1.000 | 1.000 | 1.000 | 1.000 | 1.000 | 1.000 |
| Vit B6 | 1.000 | 1.000 | 0.014 | <0.001 | 1.000 | 0.024 | <0.001 | 0.920 | 0.010 | 1.000 |
| Vit B9 | 0.004 | <0.001 | <0.001 | <0.001 | <0.001 | <0.001 | <0.001 | 0.139 | <0.001 | 0.011 |
| Vit B12 | 1.000 | 1.000 | 1.000 | 1.000 | 1.000 | 1.000 | 1.000 | 1.000 | 1.000 | 1.000 |
| Vit C | <0.001 | <0.001 | <0.001 | <0.001 | <0.001 | <0.001 | <0.001 | 0.037 | <0.001 | <0.001 |
| Vit E | 1.000 | <0.001 | 0.016 | <0.001 | <0.001 | 0.041 | <0.001 | 1.000 | 0.822 | 0.009 |
| Vit D | 1.000 | 0.287 | 0.245 | 0.121 | 1.000 | 1.000 | 1.000 | 1.000 | 1.000 | 1.000 |
| Calcium | 0.813 | 0.003 | 0.042 | 0.003 | 0.116 | 1.000 | 0.320 | 1.000 | 1.000 | 1.000 |
| Potassium | 0.636 | 0.002 | <0.001 | <0.001 | 0.290 | <0.001 | <0.001 | 0.049 | <0.001 | 0.003 |
| Iron | 1.000 | 1.000 | 1.000 | 1.000 | 1.000 | 1.000 | 0.734 | 1.000 | 1.000 | 1.000 |
| Magnesium | 1.000 | 1.000 | 1.000 | 0.699 | 1.000 | 1.000 | 0.431 | 1.000 | 1.000 | 1.000 |
| Phosphorus | 1.000 | 0.765 | 0.477 | 0.316 | 1.000 | 0.802 | 0.542 | 1.000 | 1.000 | 1.000 |
| Zinc | 1.000 | 1.000 | 1.000 | 1.000 | 1.000 | 1.000 | 0.382 | 1.000 | 1.000 | 1.000 |
| Copper | 1.000 | 1.000 | 1.000 | <0.001 | 1.000 | 1.000 | 0.080 | 1.000 | 0.023 | 1.000 |
| Iodine | 1.000 | 0.101 | 0.391 | 0.003 | 0.064 | 0.374 | 0.010 | 1.000 | 1.000 | 1.000 |
| Selenium | 1.000 | 1.000 | 1.000 | 0.753 | 1.000 | 0.353 | 0.006 | 1.000 | 0.361 | 1.000 |
| SFA | 1.000 | 1.000 | 1.000 | 0.193 | 1.000 | 1.000 | 0.020 | 1.000 | 0.027 | 0.840 |
| Sodium | 1.000 | 0.235 | 0.129 | 0.003 | 1.000 | 1.000 | 1.000 | 1.000 | 1.000 | 0.790 |
| Free sugars | 1.000 | 1.000 | 0.264 | <0.001 | 1.000 | 0.530 | <0.001 | 1.000 | 0.044 | 0.214 |
| PEF Score | 1.000 | 1.000 | 1.000 | 1.000 | 1.000 | 1.000 | 1.000 | 1.000 | 1.000 | 1.000 |
| Greenhouse gas emissions | 1.000 | 0.858 | 1.000 | 0.046 | 1.000 | 1.000 | 0.014 | 1.000 | 1.000 | 1.000 |
| Ozone depletion | 1.000 | 0.639 | 0.209 | 0.011 | 1.000 | 1.000 | 0.149 | 1.000 | 0.936 | 1.000 |
| Fine particulate matter | 1.000 | 1.000 | 1.000 | 0.023 | 1.000 | 1.000 | 0.033 | 1.000 | 1.000 | 1.000 |
| Water use | 1.000 | 0.001 | <0.001 | <0.001 | 0.021 | <0.001 | <0.001 | 0.184 | 0.020 | 1.000 |

Supplemental Table 4. Absolute (i.e., unadjusted) daily quantities and environmental impacts in each quintile of FV consumption.

| **Unit** | **Food group** | **Q1** | **Q2** | **Q3** | **Q4** | **Q5** | ***p*-value** | ***p-*trend** |
| --- | --- | --- | --- | --- | --- | --- | --- | --- |
| Total quantity (g/day) | Fruit & vegetables (incl compotes & juices) | 93.3403 | 208.18 | 327.04 | 427.27 | 633.76 | <.0001 | <.0001 |
|  | Cereals | 184.97 | 167.89 | 178.22 | 191.07 | 194.49 | 0.1982 | 0.2376 |
|  | Pulses, potatoes & nuts | 71.0254 | 59.1777 | 39.3442 | 59.1586 | 54.7746 | 0.0001 | 0.2322 |
|  | Meat, fish, eggs & alternatives | 142.00 | 145.12 | 139.28 | 151.00 | 156.52 | 0.4135 | 0.2125 |
|  | Dairies | 129.79 | 166.15 | 207.07 | 190.20 | 220.25 | <.0001 | <.0001 |
|  | Starters and mixed dishes | 191.38 | 246.42 | 259.63 | 277.76 | 313.88 | <.0001 | <.0001 |
|  | Sweetened products | 105.72 | 114.57 | 108.28 | 110.45 | 98.4384 | 0.3154 | 0.3679 |
|  | Fats | 16.5421 | 15.9122 | 15.6593 | 16.9742 | 18.8070 | 0.1886 | 0.2065 |
|  | Drinks | 1546.16 | 1627.82 | 1672.49 | 1573.37 | 1717.95 | 0.0996 | 0.0734 |
|  | Others | 23.4636 | 25.8478 | 25.4912 | 27.2314 | 23.0421 | 0.5718 | 0.9506 |
| Climate change | Fruit & vegetables (incl compotes & juices) | 0.08717 | 0.1738 | 0.2746 | 0.3650 | 0.5147 | <.0001 | <.0001 |
|  | Cereals | 0.1969 | 0.1781 | 0.1975 | 0.2060 | 0.1956 | 0.3969 | 0.4610 |
|  | Pulses, potatoes & nuts | 0.08099 | 0.06536 | 0.04592 | 0.05880 | 0.05824 | 0.0047 | 0.0447 |
|  | Meat, fish, eggs & alternatives | 2.1170 | 2.2979 | 2.0988 | 2.3970 | 2.1113 | 0.6443 | 0.8363 |
|  | Dairies | 0.2971 | 0.3812 | 0.4591 | 0.4587 | 0.5163 | <.0001 | <.0001 |
|  | Starters and mixed dishes | 0.8855 | 1.0328 | 0.9439 | 0.8345 | 0.8132 | 0.1778 | 0.1185 |
|  | Sweetened products | 0.3612 | 0.4055 | 0.3809 | 0.3548 | 0.3327 | 0.3918 | 0.2134 |
|  | Fats | 0.07276 | 0.06791 | 0.07108 | 0.08148 | 0.08342 | 0.1682 | 0.0962 |
|  | Drinks | 0.7335 | 0.8359 | 0.8415 | 0.7740 | 0.8074 | 0.6197 | 0.4438 |
|  | Others | 0.09984 | 0.1132 | 0.08385 | 0.1197 | 0.06074 | 0.0038 | 0.2278 |
| Ozone depletion | Fruit & vegetables (incl compotes & juices) | 0.01247 | 0.02638 | 0.04059 | 0.05294 | 0.07764 | <.0001 | <.0001 |
|  | Cereals | 0.01556 | 0.01412 | 0.01539 | 0.01609 | 0.01601 | 0.2832 | 0.2865 |
|  | Pulses, potatoes & nuts | 0.008197 | 0.006683 | 0.004575 | 0.005903 | 0.005741 | 0.0036 | 0.0381 |
|  | Meat, fish, eggs & alternatives | 0.1462 | 0.1439 | 0.1388 | 0.1479 | 0.1525 | 0.8288 | 0.5914 |
|  | Dairies | 0.01961 | 0.02713 | 0.03264 | 0.03275 | 0.03689 | <.0001 | <.0001 |
|  | Starters and mixed dishes | 0.07199 | 0.08380 | 0.08335 | 0.06704 | 0.06772 | 0.1151 | 0.1957 |
|  | Sweetened products | 0.01659 | 0.01943 | 0.01875 | 0.01949 | 0.01876 | 0.4435 | 0.2339 |
|  | Fats | 0.007133 | 0.006907 | 0.008287 | 0.007249 | 0.009272 | 0.0153 | 0.0197 |
|  | Drinks | 0.3433 | 0.3454 | 0.3287 | 0.3008 | 0.2854 | 0.0510 | 0.0041 |
|  | Others | 0.006835 | 0.007608 | 0.007180 | 0.008319 | 0.006672 | 0.4617 | 0.8869 |
| Fine particulate matter | Fruit & vegetables (incl compotes & juices) | 0.006017 | 0.01211 | 0.01918 | 0.02504 | 0.03580 | <.0001 | <.0001 |
|  | Cereals | 0.01491 | 0.01388 | 0.01524 | 0.01582 | 0.01544 | 0.3877 | 0.2264 |
|  | Pulses, potatoes & nuts | 0.005030 | 0.004095 | 0.002961 | 0.003656 | 0.003970 | 0.0101 | 0.0941 |
|  | Meat, fish, eggs & alternatives | 0.2397 | 0.2487 | 0.2334 | 0.2663 | 0.2427 | 0.7899 | 0.6021 |
|  | Dairies | 0.02292 | 0.02811 | 0.03421 | 0.03453 | 0.03935 | <.0001 | <.0001 |
|  | Starters and mixed dishes | 0.09509 | 0.1104 | 0.1033 | 0.08585 | 0.08195 | 0.0752 | 0.0395 |
|  | Sweetened products | 0.02322 | 0.02695 | 0.02507 | 0.02405 | 0.02105 | 0.0786 | 0.1412 |
|  | Fats | 0.005528 | 0.005428 | 0.005954 | 0.006545 | 0.007162 | 0.0360 | 0.0061 |
|  | Drinks | 0.07933 | 0.09035 | 0.09066 | 0.08388 | 0.09099 | 0.5343 | 0.2132 |
|  | Others | 0.009414 | 0.01058 | 0.007769 | 0.01105 | 0.005720 | 0.0043 | 0.2153 |
| Water use | Fruit & vegetables (incl compotes & juices) | 0.7479 | 1.6784 | 2.8289 | 4.0476 | 5.0890 | <.0001 | <.0001 |
|  | Cereals | 1.3379 | 1.1782 | 1.5249 | 1.5329 | 1.1060 | 0.3867 | 0.8146 |
|  | Pulses, potatoes & nuts | 0.1251 | 0.1272 | 0.08939 | 0.1335 | 0.1536 | 0.0147 | 0.1129 |
|  | Meat, fish, eggs & alternatives | 0.9408 | 0.9689 | 0.9539 | 0.9565 | 1.0799 | 0.6941 | 0.2989 |
|  | Dairies | 0.1221 | 0.1510 | 0.1823 | 0.1816 | 0.1981 | <.0001 | <.0001 |
|  | Starters and mixed dishes | 0.7135 | 0.8546 | 0.9450 | 0.8450 | 0.8394 | 0.3650 | 0.3214 |
|  | Sweetened products | 0.3820 | 0.5374 | 0.4383 | 0.5282 | 0.4904 | 0.0549 | 0.1899 |
|  | Fats | 0.06446 | 0.06790 | 0.1010 | 0.08849 | 0.1368 | <.0001 | <.0001 |
|  | Drinks | 0.6804 | 0.6802 | 0.6527 | 0.5799 | 0.6350 | 0.0433 | 0.0748 |
|  | Others | 0.09456 | 0.1024 | 0.09814 | 0.1109 | 0.09605 | 0.8205 | 0.7564 |
